# The prevalence of left ventricular hypertrophy associated with type-2 diabetes in Shiraz, Iran: a cross-sectional study

**DOI:** 10.1186/s12872-023-03083-4

**Published:** 2023-02-15

**Authors:** Nader Parsa, Mohammad Moheb, Mohammad Javad Zibaeenezhad, Ali Karimi-Akhormeh, Maurizio Trevisan, Lisa Wallin, Pari Mahlagha Zaheri, Mehrab Sayadi, Iman Razeghian-Jahromi, Alireza Moaref

**Affiliations:** 1 Cardiovascular Research Center, Shiraz University of Medical Sciences, 3rd Floor, Mohammad Rasoolallah Research Tower, Khalili St., Shiraz, 71936-35899 Iran; 2 City College of New York Provost & Senior Vice President for Academic Affairs, Dean of Medical School, New York, USA; 3 College of Health Sciences, Vin University, Hanoi, Vietnam; 4 Strong Memorial Hospital, University of Rochester, Rochester, NY USA; 5 Shiraz University of Medical Sciences, Shiraz, Iran

**Keywords:** LVH, CVD, T2DM, ECG

## Abstract

**Background:**

Left ventricular hypertrophy (LVH) is a common diagnosis in patients with cardiovascular disease (CVD). The prevalence of LVH among patients with Type-2 Diabetes Mellitus (T2DM), high blood pressure and aging is higher than the healthy population and has been independently associated with an increased risk for future cardiac event, including stroke. The aim of this study is to identify the prevalence of LVH among T2DM subjects and evaluate its association with related risk factors of CVD patients in the metropolis of Shiraz, Iran. The novelty of this study is that there has been no known published epidemiological study related to the relationship of LVH and T2DM on this unique population.

**Materials and method:**

This cross-sectional study was designed based on collected data of 7715 free dwelling subjects in the community-based Shiraz Cohort Heart Study (SCHS) from 2015 to 2021, ages 40–70 years. Overall, 1118 subjects with T2DM were identified in the SCHS and after exclusion criteria, 595 subjects remained eligible for study. Subjects with electrocardiography (ECG) results, which are appropriate and diagnostics tools, were evaluated for the presence of LVH. Thus, the variables related to LVH and non-LVH in subjects with diabetes were analyzed using version-22 statistical package for social sciences software program to ensure consistency, accuracy, reliability, and validity for final analysis. Based upon related variables and identifying LVH and non-LVH subjects, the relevant statistical analysis was implemented to ensure its consistency, accuracy, reliability, and validity for final analysis.

**Results:**

Overall, the prevalence of diabetic subjects was 14.5% in the SCHS study. Furthermore, the prevalence of hypertension in the study subjects aged 40–70 years was 37.8%. The prevalence of hypertension history in T2DM study subjects for LVH compared to non-LVH was (53.7% vs. 33.7%). The prevalence of LVH among patients with T2DM as the primary target of this study was 20.7%. Analytical findings comparing both LVH and non-LVH subjects who have T2DM identified significance for variables in the older (≥ 60) mean and categorical age group (*P* < 0.0001), history of hypertension (*P* < 0.0001), mean and categorical duration of hypertension in years (*P* < 0.0160), status of controlled versus uncontrolled hypertension level (*P* < 0.0120), the mean systolic blood pressure (*P* < 0.0001) as well as mean duration years of T2DM and categorical duration of diabetes in years (< 0.0001 and *P* < 0.0060), mean fasting blood sugar (< 0.0307) and categorical status of FBS Level (mg/dl): controlled and uncontrolled FBS status of controlled vs. uncontrolled FBS levels *P* < 0.0020). However, there were no significant findings for gender (*P* = 0.3112), diastolic blood pressure mean (*P* = 0.7722) and body mass index (BMI) mean and categorical BMI (*P* = 0.2888 and *P* = 0.4080, respectively).

**Conclusion:**

The prevalence of LVH in the study increases significantly among T2DM patients with hypertension, older age, years of hypertension, years of diabetes, and higher FBS. Thus, given the significant risk of diabetes and CVD, evaluation of LVH through reasonable diagnostic testing with ECG can help reduce the risk of future complications through the development of risk factor modifications and treatments guidelines.

## Introduction

Etiologically, various clinical conditions can lead to left ventricular hypertrophy (LVH). The leading cause of LVH is hypertension. The severity of LVH is affected by the severity of the hypertension along with the duration and course, aging, race, gender, history of diabetes, stroke and so on. When the heart is exposed to high overload from uncontrolled hypertension this causes more concentric remodelling and concentric hypertrophy [1] that has a direct association with LVH [2–4]. Pathophysiological LVH or an increase in myocardial muscle mass, is caused by an enlarged cardiomyocyte. The complex chain of cellular mechanisms includes electrophysiological and functional processes that affects all types of heart cells [5]. However, the most important feature of physiological hypertrophy is that ventricular function (both systolic and diastolic function) remains normal or even improved rather than impaired. Cardiac fibrosis is initially manifested by diastolic dysfunction, although systolic dysfunction occurs in the later stages [6]. Furthermore, epidemiological studies of prevalence of T2DM is growing rapidly, with the number of patients worldwide rising from 135 million in 1995 to 422 million in 2014 and expected to reach 700 million by 2040. Therefore, diabetes has become a major health problem that has attracted worldwide attention [7]. Diabetes increases the risk of complications related to small and large vessels, heart muscle tissue and causes changes in the heart tissue, pathological enlargement, LVH and coronary artery disease (CAD) [8]. Left ventricular (LV) enlargement of the heart is the most important and prominent feature in the heart of diabetic patients. T2DM, in addition to left ventricular hypertrophy, also affects the left atrium (LA) and increases its volume. Following these structural changes in LA and LV, cardiac activity is also affected and causes LV systolic and diastolic dysfunction. Patients with T2DM are much morelikely to develop CVD than the general population.

Epidemiologic prevalence studies of LVH in various communities, especially in the United States (US) used different methods, including ECG, echocardiography (ECHO), or Cardiac Magnetic Resonance Imaging (CMRI), or a combination of each of these three, to diagnose LVH. The accuracy of diagnosis was dependent on the diagnostic tools used to confirm LVH and severity [9]. Various global studies estimate LVH in the general population at 35.6% and the prevalence of LVH in people with severe hypertension is 77% [10]. Data from the US Multi-Ethnic Study of Atherosclerosis (MESA) show that the prevalence of LVH based on ECG in the general population was 6.1% and those with diabetes was 10.4% [11, 12]. Other studies from different countries estimate LVH is 35.6% and with severe hypertension is 77% [10].

Epidemiological, clinical and laboratory findings show a strong association between T2DM and cardiac muscle tissue abnormalities, such as diabetic cardiomyopathy in patients with no risk factors such as hypertension, no alcohol use or CAD [13].

A study conducted by Jorgensen PG, et al. on 1,030 T2DM cohort subjects using ECHO found that 49.8% (513) were abnormal, of which 19.4% (178) had left ventricular diastolic dysfunction (LVDD) and 21% (213) had LVH and 19.6% (200) had left atrial enlargement. The LVH prevalence increased markedly with age from 31.1% in the youngest group (< 55 years) to 73.9% in the oldest group (> 75 years) **(***P* < 0.001**)** and was equally distributed among the genders (*P* = 0.76) [14].

The study by Jobe M, et al. on 534 individuals with a history of diabetes evaluated based on ECG diagnostic criteria identified 35.2% of diabetic patients have LVH based on at least one of the diagnostic criteria and the prevalence of LVH was 24.8% among males and 39.5% in females. Among them 66.4% with LVH had a history of hypertension (HTN) due to increased systolic and diastolic blood pressure taken from individuals during the study which was significantly associated with the prevalence of LVH but not significant for BMI [15].

A study by Lutale JJ, et al. on 237 diabetic patients with ECG-LVH criteria had significantly higher systolic blood pressure (SBP) and mean blood pressure values than patients without LVH-ECG although prevalence of LVH among T2DM patients that was 15.5%. In logistic regression analysis, there was significant association in subjects of both genders, age, duration of diabetes, waist circumference, higher SBP, and diastolic blood pressure as independent variables. It was notable, for every 10-mmHg increase in SBP, the chance of developing LVH increased by 15% [16].

Bruno G, et al. studied 965 subjects with T2DM to evaluate the effect of gender on the incidence of LVH and found 17.1% (165) diabetic subjects had LVH. After adjusting for age, hypertension and BMI results they identified the incidence of LVH were significantly higher among females compared to males (23.5% vs. 8.4%). The prevalence of hypertension in study subjects was 83.9% with no significant differences with ECG-LVH than those without LVH (86.1% vs. 83.5%, *P* = 0.41). There were no significant findings for ECG-LVH in subjects with BMI categories < 26, 26–30, > 30 for different genders (*P* ≥ 0.58) [17].

Authors conducted a study on the rural population of Africa to evaluate LVH and its relationship with other factors. In this study, ECGs of 539 people were evaluated and the prevalence of ECG-LVH was 16.4% with a significantly higher prevalence in males than females (20.4% vs. 8.2%, *P* = 0.001).

Prevalence of diabetes and hypertension were 3.7% and 35.4%. There was a high prevalence among middle- and older age groups and the lowest prevalence was seen at the young age group in both genders (P-value < 0.0001). Additionally, there was a significant relationship between LVH with high blood pressure (systole and diastole) (*P* < 0.001) [18].

The Estes EH, et al. study in the United States analysed a total of 14,984 participants through follow-up for a duration of 21.7 years, from the Atherosclerosis Risk in Communities (ARIC) cohort using the Romhilt-Estes criterion (any limb R wave or S wave ≥ 2.0 mV (20 mm); QR S in V1 or S in V2 ≥ 3.0 mV (30 mm); QR R in V5 or R in V6 ≥ 3.0 mV (30 mm) a score of 5 or more indicates “definite” LVH; a score of 4 indicates “probable” LVH) to determine the prevalence of LVH. They found that 22% of the ARIC cohort had LVH. In this group 63% had diabetes and hypertension with the LVH with 44% having high systolic hypertension. In this study according to the Kruskal–Wallis analysis, LVH association with age, body mass index, systolic blood pressure, African-American ethnicity, female gender, education level, smoking, diabetes, total cholesterol, hypertension, use of antihypertensive drugs (except a family history of CHD and statin use), were highly significant (*P* < 0.0001) [19].

Chang-Min Chung et al. conducted a study to evaluate the prevalence of LVH in patients with HTN based on Romhilt-Estes criteria. From a total of 984 patients 128 (13%) had LVH. They found that 72% of people with LVH and 52% of non-LVH participants were males (*P* < 0.001). The prevalence of diabetes was 30% in those with LVH and 26% without LVH (*P* ≥ 0.429). BMI was 25.6 ± 3.3 in the group with LVH and 27.3 ± 11.1 in the group without LVH (*P* ≥ 0.101). There were no significant differences in terms of diabetes mellitus, smoking status, high blood lipids, coronary artery disease, BMI, total cholesterol, triglycerides, HDL, LDL, renal function, haemoglobin, and c-reactive protein. Comparing LVH group and non-LVH in males, results identified younger subjects having significantly lower systolic and diastolic blood pressure (*P* < 0.001) and lower pulse pressure (*P* < 0.001) [20].

A study in China examined 455 patients with T2DM for 4.7 years, in which FBS and hemoglobin A 1c (HbA1c) levels were measured from blood, and cardiac indices were measured by echocardiography. The results showed that increased FBS was independent and significantly associated with changes in the left ventricle (β = 0.137; *P* = 0.031), interventricular septum (β = 0.215; *P* = 0.001), left ventricular posterior wall thickness (β = 0.129; *P* = 0.048), and left ventricular mass index (β = 0.227; *P* < 0.001). After stratified by mean HbA1c levels, coefficient of variation-fasting plasma glucose (CV-FPG) was still independently associated with high parameters in patients with HbA1c ≥ 7% [21].

Another study was performed on 3,179 cases in which individuals were divided into 4 groups: normal blood sugar, impaired fasting blood glucose, patients with T2DM less than 15 years, and patients with T2DM more than 15 years. The group with diabetes more than 15 years were associated with the worst LVH (*P* < 0.0001). Results showed that the older people with diabetes, were at a higher risk of developing LVDD, LVH and left ventricular systolic dysfunction (LVSD) [22].

A study conducted in Nigeria on 122 T2DM patients with a mean age of 55 years, the average length of their illness was 5.1 years. Results showed that the diameter of the ventricular wall of T2DM patients in the diastole phase increased significantly compared to the control group with 27.8%, which is highly significant. Also, 50.8% of T2DM people had some form of LVH [23].

Therefore, the novelty of any study within various societies is specific to the social conditions, lifestyle, and culture in relation to illness that are subjected to the similar disease of the research, diverse sample size, socio-demographic risks factors, and normally distributed throughout the study subjects of that comprehensive society**.**

Therefore, based on previous studies in other countries and the rapidly growing prevalence of T2DM in Iran, we decided to investigate the prevalence of LVH in diabetic patients as an independent risk factor in cardiovascular disease in the SCHS, Shiraz, Iran. The population of Shiraz, Iran is comprised of many different groups (tribal, clans, migrants, immigrants) with genetic diversity who have settled in the larger metropolis in and around the city of Shiraz subjected to changing lifestyles (rural to urban, industrialization) that have impacts on health secondary to LVH, diabetes and cardiovascular diseases that have not been researched and published. Thus, it is hoped that the findings of this research study on this diverse population will be novel and help in planning and development of comprehensive health programs.

Thus, the aim of this study is to evaluate the prevalence of LVH based on electrocardiography and related risk factors in subjects with T2DM in the SCHS, in Shiraz.

## Method and material

This cross-sectional study was designed based on collected data of 7715 free dwelling subjects in the community-based Shiraz Cohort Heart Study (SCHS) from 2015 to 2021 aged 40–70 years old who reside in the metropolis of Shiraz, Iran. In this study those subjects with condition of left and right bundle branch block, existence of flutter rhythms and atrial fibrillation, distorted ECG, lack of ECG, patients with renal dialysis and subjects with a history of hospitalization related to myocardial infarction were excluded**.** After the exclusion criteria, 595 subjects with T2DM were eligible for statistical analysis.

Diagnosis of LVH in this study was based on ECG which was performed at each exam with a standard supine 12-lead resting ECG to evaluate for ventricular hypertrophy. The main ECG changes associated with ventricular hypertrophy are increases in QRS (Q-wave, R-wave and S-wave in the ECG represent ventricular depolarization) height and duration, changes in QRS immediate and mean vectors (deviation of the heart axis to the left), abnormalities in the ST and T-waves, and abnormalities in the P-wave. These changes correlated with direct or indirect assessments of ventricular size or mass to establish on ECG criteria for the diagnosis of hypertrophy*.* Different diagnostic criteria have been proposed based on ECG changes. In terms of LVH diagnostic criteria for LVH are based on measurements of QRS voltages. QRS voltages are affected by factors other than left ventricular size or mass. These factors include age, gender, race, and body type and so on.

The common feature of all of them is high specificity and low sensitivity. The following 3 well-known criteria are commonly used; Romhilt-Estes [24], Sokolow-Lyon [11] and Cornell Voltage [25] to assess the presence of LVH on the ECG. In this study, mainly the Romhilt-Estes diagnostic criterion was used. If this criterion is negative, the two other criteria are used to diagnose LVH.

In order to ensure consistency, accuracy, reliability, and validity the analysis was performed using the Version-22 program according to hypertension, diabetes, use of antihypertensive medications**,** blood sugar and ECGs interpretation along with other related information. The study group were between 40–70 years old, and the ages were categorized (< 50, 50–59, ≥ 60 years old) and the prevalence of LVH in each group was determined. Moreover, these results were compared separately and the relationship between aging and increasing the prevalence of LVH were accomplished.

Subsequently, those with T2DM, history of hypertension (Yes, No), and the duration of hypertension were categorized in years (< 5, 5–15, > 15), status of controlled and uncontrolled hypertension, and those subjects with diabetes with LVH and non-LVH were analysed. According to the American Diabetes Association guidelines [26], systolic blood pressure of < 140 mmHg and diastole < 90 mmHg are considered controlled, however, ≥ 140 mmHg and ≥ 90 mmHg are considered as uncontrolled blood pressure in diabetic patients [27].

In addition, duration of diabetes in the study subjects were categorized (< 5, 5–15, > 15) in years. Moreover, the years of diabetes and the relationship between the prevalence of LVH and the increase in the number of years of diabetes were investigated.

The BMI in T2DM patients, according to the World Health Organization (WHO) guide for Asia people was categorized (< 23, 23–27.5, > 27.5) in relationship with prevalence of LVH [28].

The FBS assessment related to LVH was < 130 mg/dl as controlled and ≥ 130 mg/dl as uncontrolled in diabetic study subjects [12, 26].

### Statistical methods for data analysis

Different statistical procedures were implemented for data analysis. The statistical methods used were independent *t-test* and chi-square test (*χ*^2^) or Fisher's exact test at a significant level of 5% for results and to describe the data, central indices mean, and standard deviation were used for quantitative data. Therefore, for normality related to distribution of quantitative variables, non-parametric tests such as Mann–Whitney test were used for more accurate results. For qualitative data the amount and percentage were used. Further analysis depending on the data conditions were used. All tests are performed at a 5% significant level.

### Ethics

The Ethics Committee of the Shiraz University School of Medicine required that all participants in the SCHS study receive a written consent form and pledged that all personal information be confidential.

### Data collection tools and process

The SCHS data was collected in sections: sociodemographic, medical history, anthropometric, biochemical characteristics, and clinical information. Questions related to the first and second sections were completed by well-trained interviewers with study subjects. The third to fifth parts of the questionnaire were completed by expert clinical staff through measuring and collecting information related to variables toward to study aim.

### Patient and public involvement

Members of the public were not involved in the design and conduct of the study.

## Results

Of the original population, after quality assurance and quality control (QA-QC) and testing, 595 T2DM subjects remained eligible for analysis. Of these 53.8% (320) were female and 46.2% (275) were male. In this study the prevalence of diabetes among the study subjects aged 40–70 years with LVH and high blood pressure was determined. Statistical analysis identified 20.7% (123) LVH and 79.3% (472) non-LVH subjects in this study.

Table 1 elaborates the results of means for age (years), systolic blood pressure (SBP) and diastolic blood pressure (DBP) in (mmHg), body mass index (BMI), duration (years) of hypertension, duration (years) of T2DM and fasting blood sugar (FBS) in (mg/dl) among LVH compared to non-LVH study subjects. Consequently, these results identified significant means differences related to age (*P* < 0.001), SBP (*P* < 0.001), years of hypertension (*P* < 0.04), years of diabetes (*P* < 0.001) and FBS (*P* < 0.03) in LVH compared to non-LVH subjects. However, these relationships had no significant differences for DBP and BMI (*P* = 0.769, *P* = 0.28 respectively) in LVH compared to non-LVH study subjects.

**Table 1:** Characteristics of 595 T2DM subjects for LVH and non-LVH in related to sociodemographic and clinical variables

Variable	LVH Mean ± SD 20.7% (123)	non-LVH Mean ± SD 79.3% (472)	*P* value
Age (year)	59.15 ± 7.05	54.90 ± 8.00	< 0.001
Systolic Blood Pressure (SBP)	131.24 ± 15.15	125.96 ± 12.47	< 0.001
Diastolic Blood Pressure (DBP)	79.80 ± 8.98	79.52 ± 9.69	0.769
Body Mass Index(BMI)	29.49 ± 5.36	28.99 ± 4.45	0.28
Duration Years of hypertension	8.33 ± 6.51	5.80 ± 5.76	< 0.04
Duration Years of T2DM	7.30 ± 6.39	5.16 ± 5.47	< 0.001
Fasting blood sugar (FBS)	149.92 ± 57.76	138.55 ± 50.23	< 0.03

Results in Table 2 identified 49.6% LVH subjects are females compared to 50.4% males. In addition, 54.9% females compared to 45.1% males are non-LVH. Therefore, in this study the percentage of LVH in both genders was nearly identical with no significant differences (*P* = 0.311).

**Table 2:** Characteristics of 595 T2DM subjects for LVH and non-LVH study in study population

Variable	LVH 20.7% (123)	non-LVH 79.3% (472)	*P-Value*
Gender			
Female	49.6% (61)	54.9% (259)	0.311
Male	50.4% (62)	45.1% (213)	
Age (year)			
< 50	12.2% (15)	25.7% (121)	< 0.001
50–59	35% (43)	43.2% (204)	
≥ 60	52.8% (65)	31.1% (147)	
History of hypertension			
Yes	53.7% (66)	33.7% (159)	< 0.001
No	46.3% (57)	66.3% (313)	
Duration of hypertension among 225 subjects with hypertension among diabetics for LVH and non-LVH (years)			
< 5	39.4% (26)	52.8% (84)	0.016
5–15	36.4% (24)	37.1% (59)	
> 15	24.2% (16)	10.1% (16)	
Status of controlled and uncontrolled hypertension (mmHg)			
Controlled < 140/90	63.4% (78)	75.0% (354)	0.012
Uncontrolled ≥ 140/90	36.6% (45)	25.0% (118)	
BMI			
< 23	9.8% (12)	7.4% (35)	0.408
23–27.5	26.2% (32)	31.8% (150)	
> 27.5	64.0% (79)	60.8% (287)	
Duration Diabetes (years)			
< 5	47.2% (58)	60.8% (287)	0.006
5–15	35.0% (43)	29.9% (141)	
> 15	17.9% (22)	9.3% (44)	
Status of FBS Level (mg/dl): controlled and uncontrolled FBS			
Controlled < 130	69.9% (86)	82.6% (390)	0.002
Uncontrolled ≥ 130	30.1% (37)	17.4% (82)	

The age classifications in the LVH subjects < 50 years old were 12.2% compared to 25.7% in non-LVH subjects and in LVH subjects, 35% compared to 43.2% in non-LVH subjects are aged (50–59). However, LVH subjects > 60 years old were 52.8% compared to non-LVH that were 31.1%. Therefore, percentage of LVH in younger diabetic subjects are less than non-LVH subjects. In contrast, the percentage of LVH in older diabetic subjects are significantly higher than those with non-LVH (*P* < 0.001).

In terms of history of hypertension, 53.7% had LVH and 33.7% were non-LVH. These differences indicate a significant relationship between history of hypertension and LVH in diabetic subjects compared to non-LVH diabetic subjects (*P* < 0.001). In addition, the percentage of all T2DM subjects with history of hypertension in this study was estimated at 37.8% (225 out of 595).

Due to duration of hypertension in subjects with LVH compared to non-LVH for different *years of* duration (< 5, 5–15 and > 15) the resulting difference was significant (*P* < 0.016). These differences found the more indicative risk for the longest period of hypertension duration (> 15) relative to shorter duration (≤ 15) in LVH compared to non-LVH (24.2% vs. 10.1%) was 2.4 times higher for LVH.

In general, results related to status of BP level identified 72.6% (432) of study subjects had controlled blood pressure (< 140/90 mmHg) however, 27.4% (163) had uncontrolled blood pressure (≥ 140/90 mmHg) in both groups. In detail, there is significant difference for controlled blood pressure (< 140/90 mmHg) and uncontrolled blood pressure related to LVH subjects compared to non-LVH subjects (*P* < 0.012).

In relation to duration of diabetes (years) among LVH subjects compared to non-LVH for different *years of* duration (< 5, 5–15, > 15) this result was significantly different (*P* < 0.006). These differences show the most indicative risk for the longest duration of diabetes (> 15) relative to shorter ones (≤ 15) in LVH compared to non-LVH (17.9% % vs. 9.3%) with 1.9 times higher risk for LVH.

Of 595 T2DM patients in this study, 80.0% (476) had FBS in the controlled range (< 130 mg/dl) and 20.0% (119) in the uncontrolled range (≥ 130 mg/dl) in both groups. In detail, there is significant difference for controlled range (< 130 mg/dl) and uncontrolled range (≥ 130 mg/dl) related to LVH subjects compared to non-LVH subjects (*P* < 0.002). Among them 58% (345) of T2DM < 5, 30.9% (184) 5–15 and 11.1% (66) > 15 years were reported with a history of diabetes. In this study, there were no significant difference in results for gender and BMI (*P* = 0.311, *P* = 0.408 respectively).

**Fig. 1:**
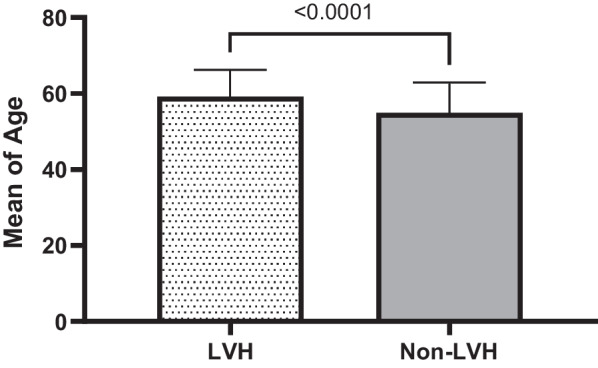
Mean ± SD of variables in LVH Compared to non-LVH subjects

**Fig. 2:**
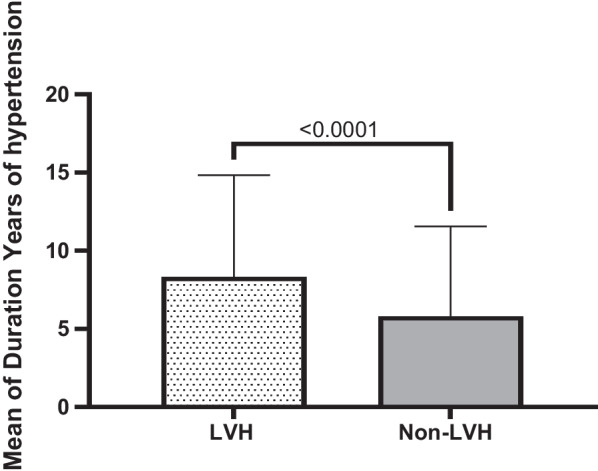
.

**Fig. 3:**
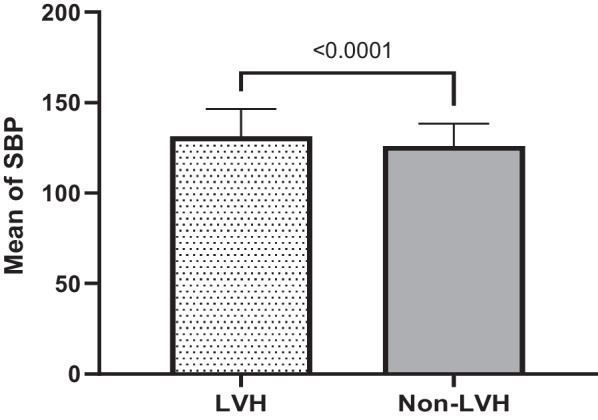
.

**Fig. 4:**
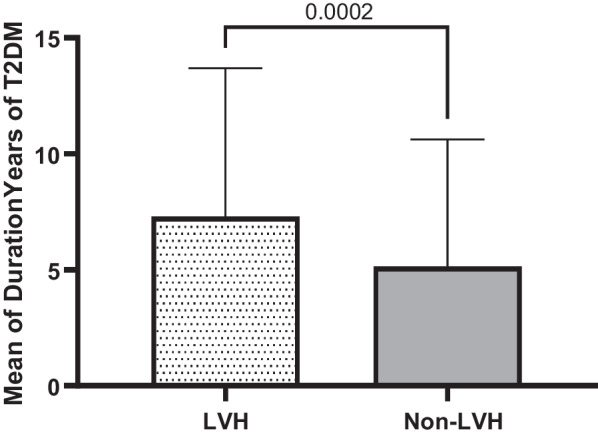
.

**Fig. 5:**
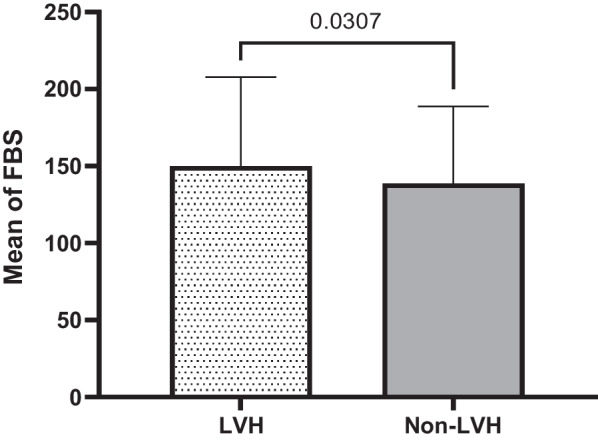
.

**Fig. 6:**
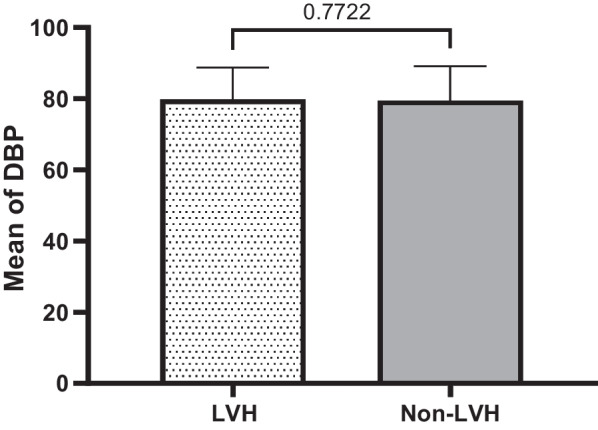
.

**Fig. 7:**
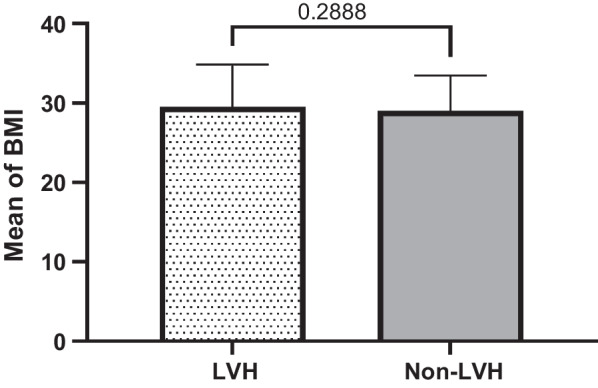
.

Quantitatively defined variables are graphically re-expressed based on mean, median and standard deviation either they are significant or not significantly associated with LVH in T2DM subjects compared to non-LVH ones. These variables include age, duration of hypertension, SBP, duration of T2DM, FBS, DBP, BMI. The results of these quantitative analysis are given in the tables (1&2) and explained in the graph of each quantitative variable in the Figs. 1, 2, 3, 4, 5, 6 and 7.

The mean age of T2DM subjects in LVH was 59.15 ± 7.05 and in the non-LVH was 54.90 ± 8.00. Therefore, the LVH subjects are older than non-LVH (Fig. 1). These results identified there is statistically significant association between aging (≥ 60) and LVH compared to non-LVH (*P* < 0.0001). The mean duration of hypertension by year among LVH, T2DM subjects and non-LVH of T2DM subjects were 8.33 ± 6.51 vs. 5.80 ± 5.76. Therefore, the LVH subjects’ duration years of hypertension was higher than non-LVH (Fig. 2). These results identified there is significant association between years of hypertension and LVH compared to non-LVH (*P* < 0.0001).

The mean SBP in the LVH subjects is higher (131.24 ± 15.15 mmHg) than non-LVH (125.96 ± 12.47 mmHg). Therefore, increased in SBP identified, the mean and median in T2DM subjects with LVH was significantly higher and associated with LVH compared to non-LVH (*P* < 0.0001) (Fig. 3). The mean duration of T2DM by year among LVH T2DM subjects and non-LVH of T2DM subjects were 7.30 ± 6.39 vs. 5.16 ± 5.47. Therefore, the LVH subjects’ years of T2DM was higher than non-LVH (Fig. 4). These results identified there is significant association between years of T2DM and LVH compared to non-LVH (*P* < 0.0002). The mean of fasting blood sugar (FBS) among LVH T2DM subjects and non-LVH of T2DM subjects were 149.92 ± 57.76 vs. 138.55 ± 50.23. Therefore, FBS level among the LVH subjects was higher than non-LVH (Fig. 5). These results identified there is significant association between hyperglycemia or higher FBS level and LVH compared to non-LVH (*P* = 0.0307). The mean DBP in the LVH T2DM subjects is almost the same as non-LVH (79.80 ± 8.98 mmHg) vs. non-LVH (79.52 ± 9.69 mmHg). Therefore, this association was not significant for DBP with LVH subjects compared to non-LVH (*P* = 0.7722) (Fig. 6). The mean BMI in the LVH T2DM subjects is almost the same as non-LVH (29.49 ± 5.36) vs. non-LVH (28.99 ± 4.45). Therefore, this association was not significant for BMI with LVH subjects compared to non-LVH (*P* = 0.2888) (Fig. 7).

## Discussion

Left ventricular hypertrophy (LVH) is an independent risk factor in patients with cardiovascular diseases (CVDs). This risk can be diagnosed by electrocardiography (ECG) or by echocardiography (ECHO) [29].

According to the Framingham Heart Study, LVH is a condition of increase in Left Ventricular Mass (LVM) that is either due to an increase in wall thickness or due to enlargement of the left ventricular cavity or both that strongly associated with an increased risk of CVD complications and death in the general population. Usually left ventricular wall thickening occurs in response to pressure overload and chamber dilatation in response to volume overload [10, 30, 31]. The prevalence of LVH among patients with T2DM, hypertension, aging, and other related risk factors for LVH is higher than the general population [32, 33].

In this study, we applied QA-QC and Testing tools [34] to consider various possible components of related risk factors such as etiology of various clinical conditions, pathophysiology and numerous worldwide epidemiologic findings, that can lead to LVH in which increase in myocardial muscle mass based on ECG as a LVH diagnostic tool [1–10].

The prevalence of diabetic subjects in this study and with LVH were mostly males and those with non-LVH were mostly females which is like prevalence date from other studies [15, 35]. However, there were no significant differences for both genders**.** Although, in a few previous studies there were conflicting findings related to LVH, that were not reliable for gender differences [15, 36]**.** Therefore, these differences may have been associated or effected by study design, social and cultural background, or lifestyle of study subjects.

Electrocardiography has identified that LVH is independently associated with increasing risk of CVD diseases [16, 37]. Consequently, ECG as an easy and economic diagnostic procedure for LVH was implemented in this study to identify the prevalence of LVH among study subjects to evaluate its association with related risk factors that was similar to other studies [15–19].

Overall, the prevalence of diabetic subjects in the study population of Shiraz metropolis city aged 40–70 years in the SCHS was similar to another comprehensive health study by Hariri, et al. in Khuzestan, Iran [38]. The prevalence of history of hypertension in T2DM study subject related to LVH compared to non-LVH was higher, 53.7% vs. 33.7%.

The prevalence of LVH among study subjects with T2DM as the main target of this study diagnosed by ECG for the presence of LVH in 2015–2021 is similar to prior study by Bruno, et al. [17]. The prevalence of history of hypertension in LVH and non-LVH in study subjects with T2DM was also high.

Similar to previous studies, findings identified the association between LVH and variables such as age [39, 40], history of hypertension, systolic blood pressure [15, 41], duration of hypertension [42, 43], duration of diabetes [39] and FBS level [39] were all significant. However, similar to other studies, there is no significant association between LVH and gender [15, 36], diastolic blood pressure [39], and BMI [15].

For more clarification of variables related to LVH, in comparison with non-LVH, these findings were elaborated in conceptual perception.

Therefore, in this community-based cohort study, age categories show that LVH prevalence was highest in the subjects older than 60 years and longer duration in years of diabetes. Results showed the history of hypertension, duration of hypertension and elevated systolic blood pressures were greater in T2DM subjects with LVH compared to non-LVH that identified a biologically plausible relation with prevalence of LVH and history of hypertension. These finding were supported by other studies [35, 42–44]. In this Shiraz study BMI status prevalence was not different in the LVH and non-LVH subjects according to WHO guide for Asia people. These findings were supported by other similar studies [15].

These findings identified biological plausibility for LVH diabetics subjects’ association with longer duration and more prevalence of LVH compared to non-LVH highest in LVH than non-LVH. Consequently, the association of diabetes duration and LVH in mean and category of diabetes duration findings were statistically significant. This study was supported by other similar studies [39].

Most of the T2DM patients in this study had controlled FBS, diabetes less than 15 years. Of those subjects who had LVH they had a longer duration of diabetes and uncontrolled FBS than subjects with non-LVH.

These findings were supported by other studies [15, 45].

## Conclusion

The prevalence of ECG documented LVH in the SCHS with T2DM study was 20.7%. The significant findings of this study included age, history of hypertension, duration of hypertension, systolic blood pressure, uncontrolled hypertension, duration of diabetes and uncontrolled fasting blood sugar related to LVH are important issues that were found to be significantly correlated with the development of CVDs.

Therefore, there is an urgent need to undertake risk factor modification, amongst others elicited in this study while developing a more comprehensive health package for the entire population of Shiraz city. Moreover, the health system could target high-risk groups by timely screening for CVD and greatly reduce the financial and human burden of disease on the community and health system. Diagnosis of LVH has increased in recent years and can be reduced and possibly reversed with treatment, thus preventing, or delaying adverse clinical outcomes of LVH.

It is hoped that this cross-sectional study will be the basis for allocation of funding to write a comprehensive risk modification program for health management in the city of Shiraz through the Ministry of Health.

### Strengths

The strengths of the SCHC study were that data are recent and solidly collected in specialized university sponsored clinics representing a sampling of adults over 40 years of age in the large metropolitan city of Shiraz, Iran. The reliability diagnostic tool of ECG is a well-known economic device with relatively low sensitivity and high specificity and fairly accurate for screening and diagnosing LVH in a large population. The ECG findings in this study were from ECGs recorded by specially trained technicians, using more sensitive recording devices than those in the usual hospital or outpatient setting. The study results strengthen the likelihood that a set of ECG parameters can be identified and validated as a risk assessment tool, useful for guidance of the physician caring for a patient with cardiovascular disease. The findings of this study clearly imply potential usefulness of the ECG as a predictive tool in clinical care of patients with cardiovascular disease.

### Limitations

The possible effect of less rigorous electrode placement and lower frequency response of recording instruments are unknown. Therefore, this study only examines the LVH risk that predicted the possible differential effects of the Romhilt-Estes score components on different types of CVDs events. However, components of different types of CVDs events warranted further study of its differential predictive ability for other cardiovascular events. The possible limitations in this study may include lack of accessibility to echocardiography (ECHO) studies to diagnose LVH. However, the ECG has a reliable performance for assessment and classification of LVH, with limited cost. This differences in the assessment of LVH rises from the indexing methods.

## Data Availability

The datasets generated and/or analyzed during the current study are not publicly available due to the statement of the ethics committee of Shiraz University of Medical Science but are available from the corresponding author on reasonable request.
